# Atomic Force Microscopy-Based Nanomechanical Profiling Reveals Oxidative Stress-Associated Structural Alterations and Curcumin-Associated Protection in Chicken Erythrocytes

**DOI:** 10.3390/cimb48090957

**Published:** 2026-09-19

**Authors:** Tianzhu Yu, Xiyao Yin, Jie Jiao, Zuobin Wang

**Affiliations:** 1 International Research Centre for Nano Handling and Manufacturing of China, Changchun University of Science and Technology, Changchun 130022, China; 2 Centre for Opto/Bio-Nano Measurement and Manufacturing, Zhongshan Institute of Changchun University of Science and Technology, Zhongshan 528437, China; 3 Ministry of Education Key Laboratory for Cross-Scale Micro and Nano Manufacturing, Changchun University of Science and Technology, Changchun 130022, China; 4 Jilin Provincial Key Laboratory for Multi-Information Nano Detection & Handling of Single Cells, Changchun 130022, China; 5 Joint Research Centre for Computer Controlled Nanomanufacturing, University of Bedfordshire, Luton LU1 3JU, UK

**Keywords:** atomic force microscopy, nanomechanical analysis, oxidative damage, chicken erythrocytes, curcumin

## Abstract

Chicken erythrocytes are nucleated blood cells that provide a useful model for assessing oxidative stress-related membrane injury, but the conventional hemolysis assays do not resolve single-cell nanoscale mechanical alterations. Here, we established an atomic force microscopy (AFM)-based workflow to characterize structural and nanomechanical alterations associated with 2,2′-azobis(2-amidinopropane) dihydrochloride (AAPH) exposure and curcumin-associated protection in chicken erythrocytes. Hemolysis assays were used to select a moderate injury condition and a protective curcumin dose. Confocal microscopy was used to assess filamentous actin (F-actin) organization and nuclear morphology, and short-term storage conditions were optimized to preserve fragile damaged cells before AFM measurements. The AAPH-induced concentration-dependent hemolysis, cytoskeletal disruption, and nuclear shrinkage altered erythrocyte morphology, characterized by decreased cellular dimensions and increased cell height. These treatments also affected nuclear mechanical properties, as indicated by increased indentation depth of the nuclear region and decreased apparent Young’s modulus and adhesion force at the nuclear region. Storage in Alsever’s solution at 4 °C best preserved the damaged erythrocytes for AFM analysis. Curcumin pretreatment reduced hemolysis and partially preserved cytoskeletal and nuclear morphology. AFM further showed that curcumin attenuated the nanoscale topographical and mechanical changes induced by AAPH. These results link biochemical injury, subcellular structural remodeling and single-cell nanomechanical phenotypes, and provide a quantitative AFM workflow for evaluating oxidative damage-associated structural and mechanical alterations and curcumin-associated preservation in fragile blood cells.

## 1. Introduction

Erythrocytes are the most abundant circulating blood cells and are essential for oxygen transport, acid–base balance and systemic metabolic homeostasis [1]. Because their membranes are continuously exposed to oxygen, heme iron and circulating oxidants, erythrocytes are highly vulnerable to oxidative stress. Oxidative injury can initiate lipid peroxidation, membrane-skeleton cross-linking, protein oxidation and leakage of intracellular contents. These changes reduce cellular deformability and promote hemolysis, thereby impairing microcirculatory function and oxygen delivery [2,3,4,5]. Therefore, erythrocytes provide a simple and sensitive model for evaluating oxidative stress-related membrane injury. Compared with mammalian erythrocytes, chicken erythrocytes retain nuclei and a relatively complete cytoskeletal architecture. Moreover, the retained nucleus in chicken erythrocytes enables direct assessment of nuclear mechanical alterations by AFM, providing additional information on subcellular responses to oxidative stress that cannot be evaluated from enucleated mammalian erythrocytes. This feature makes them particularly useful for connecting biochemical injury with nuclear morphology, cytoskeletal organization and single-cell mechanical phenotypes. However, most erythrocyte injury studies still rely on ensemble biochemical readouts. These readouts can quantify the extent of damage, but they cannot fully describe how individual cells remodel their membrane surface and mechanical properties during oxidative injury. Recent advances in single-cell biophysical analysis have highlighted the importance of integrating morphological and mechanical information with conventional biochemical assays to better understand heterogeneous cellular responses [6].

The erythrocyte membrane is not only a passive boundary but also a mechanically active interface that determines cell shape, deformability, adhesion behavior and resistance to external stress [6]. Once the oxidative stress damages membrane lipids and membrane-associated proteins, the spatial organization of the membrane skeleton can be altered [7]. Such alterations may change cell height, spreading area, surface roughness, local indentation response and adhesion force [8]. These parameters are closely related to erythrocyte physiological function. They are also important for distinguishing mild membrane stress from severe structural failure. For nucleated avian erythrocytes, the relationship between the nucleus, the surrounding cytoskeletal network and the membrane surface further increases the complexity of injury evaluation. A method that can evaluate morphology and mechanics at the single-cell level is therefore needed to provide more direct evidence for oxidative damage-associated structural alterations and curcumin-associated preservation.

Another reason for performing single-cell analysis is that erythrocyte oxidative damage is often heterogeneous. Under the same oxidant concentration, some cells may remain nearly intact, whereas others may show shrinkage, swelling, membrane rupture or marked mechanical weakening [9]. A mean hemolysis value cannot resolve this heterogeneity. It also cannot indicate whether the remaining intact cells have already undergone sublethal nanoscale changes. These sublethal changes are important because they may influence cell survival and mechanical function before visible hemolysis occurs. Therefore, the specific knowledge gap is the lack of direct single-cell evidence on structural and nanomechanical alterations in intact nucleated chicken erythrocytes before complete hemolysis, and on whether curcumin-associated reductions in hemolysis are accompanied by preservation of these biophysical properties.

AAPH is a water-soluble azo-compound that decomposes under physiological conditions to generate peroxyl radicals [10,11]. These radicals can initiate lipid peroxidation of membrane phospholipids, resulting in oxidative damage to membrane structures and associated cytoskeletal components. Therefore, AAPH provides a controlled model for investigating peroxyl-radical-mediated oxidative injury in erythrocytes. Because the radical generation process is controllable, AAPH is widely used to construct oxidative damage models in erythrocytes [12,13]. AAPH treatment can reproduce several key features of oxidative injury, including membrane disruption, cytoskeletal damage and hemoglobin release. For analytical studies, the most important issue is not simply to induce maximal hemolysis. Excessive hemolysis produces debris and leaves too few intact cells for imaging and force-curve acquisition. In contrast, a moderate injury model can maintain measurable damage while preserving enough structurally intact cells for downstream nanoscale analysis. Therefore, the careful selection of AAPH concentration is a necessary first step before establishing an atomic force microscopy-based analytical workflow [14].

Curcumin, a polyphenolic compound derived from *Curcuma longa*, has attracted considerable interest because of its antioxidant, anti-inflammatory and cytoprotective activities [15,16,17]. Previous studies suggest that curcumin may influence erythrocyte responses through mechanisms including radical scavenging, metal ion chelation, inhibition of lipid peroxidation and regulation of antioxidant systems [18,19]. These studies provide a useful biochemical background for interpreting structural and mechanical changes associated with curcumin treatment. Nevertheless, available evidence is still dominated by bulk assays, including hemolysis rate, malondialdehyde content and antioxidant enzyme activity [20,21]. These measurements are informative but indirect. They cannot determine whether curcumin preserves the membrane surface, maintains cytoskeletal continuity, reduces cell deformation or restores local mechanical behavior at the level of individual cells. As a result, structural and nanomechanical evidence supporting the curcumin protection remains insufficient. These limitations indicate that complementary approaches capable of directly evaluating cellular structure and mechanics are needed.

This limitation is particularly relevant when antioxidants are evaluated for membrane protection [3,4,5]. A decrease in hemolysis may indicate that fewer cells have undergone complete rupture, but it does not necessarily prove that the surviving cells have recovered normal morphology and mechanics. Some cells may remain intact while retaining abnormal stiffness, altered adhesion or increased indentation depth. These residual mechanical abnormalities may reflect incomplete protection [6,7,8,9]. Conversely, restoration of nanomechanical parameters may provide more direct evidence that the membrane–cytoskeleton system has been preserved. Therefore, evaluating curcumin protection requires a method that can go beyond hemoglobin release and examine the physical state of individual erythrocytes.

AFM provides a suitable approach to fill this gap. Unlike conventional optical imaging, AFM can obtain high-resolution surface topography and force–distance curves from the same cell under liquid and near-physiological conditions [22]. Parameters such as surface roughness, cell height, Young’s modulus, adhesion and indentation depth can be used to characterize the nanomorphological and nanomechanical states of erythrocytes [23]. These parameters can link membrane integrity with cellular mechanical functions [24]. This is particularly important for fragile cells because mechanical alterations may occur before complete lysis becomes evident. AFM has therefore become an increasingly useful tool for studying biological membranes, cell injury and drug-induced changes in cellular mechanics. In the present study, AFM is not used only as an imaging technique but also as a quantitative analytical platform for evaluating oxidative injury and antioxidant protection at the single-cell level. Recent AFM-based studies have further demonstrated its value in quantitatively characterizing cellular mechanical alterations associated with oxidative stress and membrane damage [22,23,24].

Although AFM has been applied to a variety of biological cells, its application to oxidatively damaged chicken erythrocytes still requires further methodological refinement. Many AFM studies focus on normal cells or cells that remain relatively stable during preparation [22]. By contrast, erythrocytes exposed to AAPH are more fragile and may change rapidly after treatments [10,13]. This makes the analytical process more sensitive to time, medium composition and immobilization conditions [22,23,24]. Without an optimized workflow, the measured nanomechanical differences may reflect sample degradation rather than the true oxidative damage or curcumin-mediated protection. Therefore, the present study places its emphasis on method establishment as well as biological interpretation.

Compared with conventional adherent cell models, AFM analysis of chicken erythrocytes presents additional experimental challenges. Chicken erythrocytes are suspension cells rather than naturally adherent cells, and therefore require substrate modification (such as poly-L-lysine coating) for immobilization before AFM measurement. Because the attachment mainly depends on electrostatic interactions, treated or fragile cells may become less firmly attached and can be displaced by the AFM probe during scanning, which may also increase the risk of probe contamination. Moreover, chicken erythrocytes are terminally differentiated cells that cannot be continuously passaged in vitro, making long-term culture and maintenance of a consistent cellular state difficult. After AAPH-induced oxidative injury, abundant cellular debris may be generated, further increasing the difficulty of AFM acquisition and probe cleanliness control.

Moreover, AFM analysis of oxidatively damaged erythrocytes remains technically challenging. Damaged erythrocytes can shrink, swell, rupture or detach during sample storage, immobilization and scanning. These artifacts may affect the reliability of topographic images and force-curve data. In addition, inappropriate storage media or temperature conditions may aggravate the membrane injury before AFM measurements, making it difficult to distinguish treatment-induced changes from preparation-induced artifacts [25,26]. Therefore, an AFM-compatible preservation strategy is required before meaningful nanomechanical measurements can be obtained [27].

The choice of AFM measurement conditions is also important for data interpretation [28]. The force setpoint, probe properties, liquid environment, substrate coating and measurement region can influence the indentation depth, adhesion and Young’s modulus [29,30]. For nucleated chicken erythrocytes, the nuclear region and peripheral cytoplasmic region may have different mechanical responses. If these factors are not controlled, the force-curve data from different cells or treatment groups may not be comparable [28]. Thus, a clear sampling strategy is necessary to ensure that the AFM values reflect the treatment-related differences rather than the measurement variability [31]. This aligns with the analytical focus of this study on the workflow reliability and parameter interpretability, with all analyses restricted to nuclear regions.

Confocal microscopy can complement AFM by visualizing subcellular structural alterations that cannot be fully interpreted from force curves alone [32,33]. F-actin staining provides information about the membrane-skeleton organization, while nuclear staining reflects nuclear shape and structural integrity in chicken erythrocytes [32,33,34]. Combining hemolysis assays, confocal microscopy and AFM can therefore establish a multilevel analytical framework [35]. The hemolysis assays define the biochemical extent of injury. Confocal imaging verifies cytoskeletal and nuclear remodeling. AFM further quantifies nanoscale morphology and mechanical behavior. This integrated strategy allows the oxidative injury and curcumin-associated structural and mechanical changes to be assessed from biochemical, structural and mechanical perspectives. It also helps explain why a change in hemolysis rate may correspond to changes in single-cell morphology and mechanics [33,35].

However, the unresolved issue is not simply whether oxidative stress causes erythrocyte lysis, but how individual erythrocytes undergo nanoscale structural remodeling and mechanical deterioration before or beyond detectable hemolysis, and whether antioxidant intervention can preserve these biophysical properties.

In this study, we established an AFM-based nanomechanical profiling workflow for evaluating AAPH-induced oxidative damage and curcumin-associated structural and mechanical preservation in chicken erythrocytes. The hemolysis assays were first used to select an AAPH injury level suitable for downstream AFM analysis rather than complete cell destruction. The curcumin pretreatment was then evaluated to determine a suitable condition for subsequent analyses. Confocal microscopy was used to observe F-actin organization and nuclear morphology. In parallel, short-term storage conditions for damaged erythrocytes were optimized to preserve cell integrity before AFM measurements. Finally, liquid-phase AFM-based quantitative imaging (QI) was employed to simultaneously characterize erythrocyte morphology and nuclear mechanical properties. This approach enabled the quantification of long and minor axis lengths, cell height, and nuclear nanomechanical parameters, including indentation depth, apparent Young’s modulus, and adhesion force. This work provides direct nanoscale evidence of curcumin-induced changes in erythrocyte structural and mechanical properties after oxidative challenge and offers a reproducible methodological reference for the AFM-based evaluation of oxidative stress-related injury in fragile blood cells. Compared with conventional evaluation based mainly on hemolysis, this work examines whether reduced injury is accompanied by preservation of cell structure and mechanical behavior. Such information may help improve the analytical assessment of membrane-targeted cytoprotective agents and broaden the use of AFM in fragile cell models. It also strengthens the connection between method development, parameter validation and biological application in the context of oxidative stress analysis.

## 2. Materials and Methods

### 2.1. Reagents and Materials

Curcumin (purity ≥ 95%) was obtained from Yuanye Biotechnology Ltd. (Shanghai, China). AAPH, dimethyl sulfoxide (DMSO), poly-L-lysine (molecular weight 150–300 kDa), 4% paraformaldehyde, 0.5% Triton X-100, fluorescein isothiocyanate (FITC)-labelled phalloidin, 4′,6-diamidino-2-phenylindole (DAPI) staining solution, Alsever’s solution and 20% chicken erythrocyte suspension were purchased from Solarbio Science & Technology Ltd. (Beijing, China). Phosphate-buffered saline (PBS, pH 7.4) and normal saline were of analytical grade. Deionized water was used throughout the experiments.

### 2.2. Erythrocyte Suspension

The purchased erythrocyte suspension was provided as a ready-to-use 20% (*v*/*v*) suspension in Alsever’s solution by Solarbio (catalog number: S9469). Alsever’s solution, which contains sodium citrate dihydrate, citric acid monohydrate, glucose, and sodium chloride, is an isotonic balanced salt solution used for erythrocyte preservation. The commercially supplied suspension was stored at 4 °C and used within 2 weeks. During storage, the suspension was gently resuspended once daily to avoid erythrocyte sedimentation and minimize hemolysis. Before each experiment, the suspension was gently inverted several times until homogeneous and visually inspected to confirm the absence of obvious hemolysis or aggregation. The suspension was used directly without additional centrifugation, washing, or replacement of Alsever’s solution. Three independent biological experiments were performed using aliquots from the same commercial batch to minimize batch-to-batch variation. Therefore, all experiments in this study were conducted using erythrocytes from the same commercial batch, and variation among different donor chickens was not considered in the experimental design. The potential influence of Alsever’s solution was taken into account. Because all groups, including controls and treated samples, were analyzed under the same storage medium and duration, any minor storage-related effects would be expected to influence all groups similarly rather than create treatment-specific differences.

### 2.3. AFM Substrate Coating

Poly-L-lysine-coated substrates were prepared using 35-mm cell culture dishes. Briefly, 1 mL of 0.01% poly-L-lysine solution was added to each dish to fully cover the substrate surface, followed by incubation at room temperature for 1 h. After incubation, the poly-L-lysine solution was removed and the dishes rinsed three times with deionized water using 1 mL each time. The coated dishes were then air-dried overnight under sterile conditions on a clean bench, sealed with Parafilm, and stored at room temperature until use.

### 2.4. AAPH-Induced Hemolysis Assay and Curcumin Pretreatment

Hemolysis was evaluated by measuring hemoglobin release from erythrocytes at 540 nm. All experiments were performed in 10 mL centrifuge tubes. For AAPH-induced hemolysis analysis, 400 μL of 20% chicken erythrocyte suspension was added to each tube. To maintain the same erythrocyte concentration as that used in the subsequent curcumin pretreatment experiments, 400 μL of PBS was added as the corresponding dilution control. The mixtures were preincubated at 37 °C for 1 h in a shaking incubator. Subsequently, 800 μL of AAPH working solutions with different concentrations (120, 140, 160, 180, 200, 220, and 240 mM) was added. Based on the concentration-dependent hemolysis results from the preliminary screening, 150 mM AAPH was additionally validated and selected as the oxidative stress condition for subsequent curcumin pretreatment experiments. The mixtures were incubated at 37 °C for 2 h in a shaking incubator. After incubation, the reaction mixtures were diluted with 5 mL of PBS. For the total hemolysis control, erythrocyte suspensions were treated with 5 mL of deionized water to induce complete hemolysis. Erythrocytes treated with PBS without AAPH or curcumin were used as the untreated control, while PBS was used as the blank control for background correction. The samples were centrifuged at 1300 rpm for 5 min at 4 °C, and the supernatants were collected. Subsequently, 200 μL of each supernatant was transferred into a 96-well microplate, and five replicate wells were prepared for each treatment. The absorbance was measured at 540 nm using an Epoch 2 microplate reader manufactured by BioTek Instruments, Inc. (Winooski, VT, USA). All AAPH-induced hemolysis experiments were independently repeated three times using aliquots from the same erythrocyte suspension batch, and the five replicate wells represented technical replicates within each independent experiment.

The hemolysis percentage was calculated using the following equation:

$$\mathrm{Hemolysis}(\%)=\frac{A_{\mathrm{sample}}-A_{\mathrm{PBS}}}{A_{\mathrm{total}\;\mathrm{hemolysis}}-A_{\mathrm{PBS}}}\times 100\%$$

where $A_{\mathrm{sample}}$ was the absorbance of the experimental sample, $A_{\mathrm{PBS}}$ the absorbance of the PBS blank, and $A_{\mathrm{total}\;\mathrm{hemolysis}}$ the absorbance corresponding to the complete hemolysis. The moderate injury condition was defined as an AAPH concentration that induced approximately 30–40% hemolysis, representing a balance between sufficient oxidative damage induction and preservation of enough intact erythrocytes for downstream confocal microscopy and AFM-based nanomechanical analysis. This injury level provided sufficient erythrocyte damage while maintaining sample integrity for subsequent confocal microscopy and AFM analyses.

Due to the limited water solubility of curcumin, DMSO was used as a solvent to prepare the curcumin stock solution. Curcumin was dissolved in DMSO at a concentration of 10 mM and stored in aliquots at −20 °C until further use. Before each experiment, the stock solution was diluted with PBS to prepare the curcumin working solutions at concentrations of 1.25, 2.5, 5, 10, 20, 40 and 80 μM. The curcumin concentrations stated in this study refer to the concentrations of the working solutions added to the erythrocyte suspension. For the curcumin pretreatment, 400 μL of 20% erythrocyte suspension was mixed with 400 μL of curcumin working solution and incubated at 37 °C for 1 h in a shaking incubator. Vehicle controls containing final concentrations of DMSO (0.00625%, 0.0125%, 0.025%, 0.05%, 0.10%, 0.20%, and 0.40%, corresponding to 1.25, 2.5, 5, 10, 20, 40, and 80 μM curcumin treatments, respectively) were included to exclude the effect of DMSO. The control and model groups were prepared by mixing 400 μL of 20% erythrocyte suspension with 400 μL of PBS under identical conditions. Based on the AAPH optimization described above, 150 mM AAPH working solution was selected for subsequent oxidative injury experiments. Following pretreatment, the curcumin-pretreated and model groups were treated with 800 μL of 150 mM AAPH working solution, whereas the control group received an equal volume of PBS. All mixtures were incubated at 37 °C for an additional 2 h. The experimental samples were diluted with PBS, while the complete hemolysis control was diluted with deionized water. After centrifugation, absorbance was measured at 540 nm as described above, and the hemolysis rate calculated. All curcumin pretreatment experiments were independently repeated three times using aliquots from the same erythrocyte suspension batch, and five replicate wells were prepared for each treatment condition in each independent experiment.

### 2.5. Confocal Microscopy

After treatment, erythrocytes from each group were collected and added onto poly-L-lysine-coated dishes for 30 min to allow cell attachment. The incubation solution was then removed, and the attached erythrocytes were fixed with 4% paraformaldehyde for 10 min at room temperature, followed by three washes with PBS. The cells were then permeabilized with 0.5% Triton X-100 for 5 min, followed by three PBS washes. FITC-phalloidin was used for F-actin visualization because phalloidin specifically binds polymerized F-actin and is widely applied for imaging actin filament organization in fixed cells. Therefore, the FITC fluorescence signals were interpreted as representing F-actin organization based on the specific binding property of phalloidin to polymerized F-actin. DAPI counterstaining was used to distinguish nuclear signals from F-actin fluorescence in avian nucleated erythrocytes. Subsequently, 200 μL of FITC-labelled phalloidin working solution was added to label F-actin, and the samples were incubated for 30 min at room temperature in the dark. After incubation, the cells were washed three times with PBS. Nuclear counterstaining was performed with 200 μL of DAPI staining solution (100 nM), followed by three additional PBS washes.

Fluorescence images were acquired using confocal laser scanning microscopy manufactured by Carl Zeiss AG (Oberkochen, Germany). FITC-labelled phalloidin was excited at 488 nm to label F-actin, which generated green fluorescence signals representing cytoskeletal organization, while DAPI was excited at 405 nm to stain nuclei, producing blue fluorescence signals. The fluorescence signals analyzed in this study were derived from FITC-phalloidin and DAPI staining rather than curcumin autofluorescence. Confocal images from all experimental groups were acquired using identical imaging settings, including laser power, detector gain, and acquisition parameters. The same post-processing procedures were applied to all images, and no group-specific adjustments were performed. Importantly, curcumin autofluorescence was not used for image acquisition or interpretation. The fluorescence signals analyzed in this study originated from FITC-labelled phalloidin and DAPI staining, which specifically reflect F-actin organization and nuclear morphology, respectively.

Nuclear morphology was quantified using ZEISS ZEN 3.8 software. The nuclear regions were identified based on DAPI fluorescence signals, and the software automatically calculated nuclear area for each individual cell. The measured values were exported for statistical analysis. For confocal microscopy analysis, images were acquired from 10 randomly selected microscopic fields per experimental group, and nuclear area was quantified from 50 individual cells per group. Three independent biological experiments were performed using aliquots from the same commercial erythrocyte suspension batch.

### 2.6. Damaged Erythrocyte Storage for AFM

To optimize preservation conditions for sequential AFM measurements, H_2_O_2_-treated chicken erythrocytes (10 μL) were diluted in 7 mL PBS or Alsever’s solution and stored at 4 °C or 23 °C for 8 h. Subsequently, 100 μL of each preserved sample was added to poly-L-lysine-coated culture dishes containing 2 mL Alsever’s solution and incubated for 30 min to facilitate erythrocyte attachment. Cell morphology was examined using an inverted optical microscope manufactured by Nikon Corporation (Tokyo, Japan). Storage performance was evaluated according to cell contour clarity, surface smoothness, shrinkage, swelling, rupture and debris formation. The 8 h storage period was selected because the AFM measurements were completed within this time window after oxidative treatment; therefore, it represents the practical maximum interval evaluated for preserving damaged erythrocytes before AFM acquisition.

### 2.7. AFM Measurements

The erythrocyte samples were processed according to the preservation conditions described in Section 2.6. Briefly, 2 mL of Alsever’s solution and 100 μL of preserved erythrocyte suspension were added to poly-L-lysine-coated plastic culture dishes and incubated for 30 min to allow cell adhesion. The supernatant was subsequently removed to eliminate non-adherent cells and cellular debris, followed by the addition of 2 mL of fresh Alsever’s solution. AFM measurements were immediately performed under liquid conditions.

The topography and nuclear nanomechanical properties of erythrocytes were characterized using a liquid-phase AFM system (JPK NanoWizard 4 XP, Bruker, Berlin, Germany) in QI mode at 21 °C. QI is a force-curve-based imaging mode in which an approach–retraction force–distance curve is acquired at each pixel. Therefore, the force-spectroscopy data analyzed in this study were obtained directly from the force–distance curves acquired during QI mapping rather than using a separate force-spectroscopy mode. Measurements were performed in Alsever’s solution using a Bruker MLCT-C silicon nitride cantilever (Bruker, Billerica, MA, USA). The cantilever exhibited a triangular geometry with nominal dimensions of 310 μm in length, 20 μm in width, and 0.55 μm in thickness. The nominal spring constant was 0.01 N m^−1^, and the resonance frequency in liquid was approximately 7 kHz. The cantilever was coated with a reflective gold layer, and the pyramidal tip had a nominal radius of curvature of approximately 20 nm and a height of 2.5–8.0 μm. Prior to measurements, the spring constant was calibrated using the thermal noise method, and the photodetector sensitivity was experimentally calibrated from force curves obtained on a rigid substrate. The QI mode was selected because it enables force–distance curve acquisition at each pixel while minimizing lateral tip–sample forces and allowing precise control of the normal loading force. Therefore, it is suitable for fragile oxidatively damaged erythrocytes that are prone to deformation or displacement during AFM scanning. In this study, the force-spectroscopy information was obtained directly from the approach–retraction force–distance curves acquired during QI mapping rather than from a separate force-spectroscopy mode.

The low spring constant and sharp tip geometry were selected to achieve high-resolution mapping of local mechanical properties in nucleated chicken erythrocytes. QI mode minimizes lateral tip–sample forces while allowing precise control of the normal loading force, making it suitable for fragile oxidatively damaged erythrocytes that are susceptible to deformation or displacement during scanning. Although the 20 nm tip radius may increase local stress compared with larger probes, the low loading force and shallow indentation depth applied in QI mode minimized cell deformation and potential probe-induced damage. The sharp tip was particularly suitable for resolving mechanical heterogeneity within the nuclear region, where local variations in apparent Young’s modulus and adhesion were investigated.

The scan area was set to 20 μm × 20 μm with a resolution of 128 × 128 pixels. Based on previously optimized parameters established in our laboratory, the force setpoint was maintained at 0.30 nN, with a Z length of 1300 nm and a Z speed of 25 μm s^−1^. For each experimental group, 30 erythrocytes were independently measured. Ten force-curve points were randomly selected from the nuclear region of each cell and averaged to obtain a single cell-level value. To minimize substrate interference, nanomechanical analysis was restricted to the elevated nuclear region, and the thin peripheral cytoplasmic region was excluded from force-curve analysis. In addition, a low force setpoint (0.30 nN) was used to limit indentation depth relative to the local cell height. Nevertheless, because finite-thickness effects cannot be completely excluded in cellular AFM measurements, the measured mechanical parameters were interpreted as local apparent responses under the applied AFM conditions.

Statistical comparisons were performed using the 30 independent cell-level measurements, while individual force curves were not considered independent biological replicates. Force–distance curves were analyzed to obtain the apparent Young’s modulus, adhesion force, and indentation depth using the Hertz model. Because erythrocytes are heterogeneous viscoelastic biological cells, the Hertz model was used here to obtain an apparent Young’s modulus for comparative analysis. Therefore, the AFM-derived mechanical parameters should be interpreted as local apparent mechanical responses of the erythrocyte nuclear region under the specific AFM loading conditions, rather than the intrinsic bulk elastic properties of the entire cell. All measurements were conducted under identical probe and acquisition conditions to ensure reliable comparison among groups. The cell was considered the experimental unit for AFM analysis because multiple force curves obtained from the same cell represent repeated measurements rather than independent biological replicates. Accordingly, statistical analyses were performed at the cell level after averaging repeated force-curve measurements from the same cell, and individual force curves were not treated as independent observations.

### 2.8. Statistical Analysis

Statistical analysis was performed using SPSS 27.0 (IBM, Armonk, NY, USA). One-way ANOVA, followed by Dunnett’s post hoc test, was used when each treatment was compared with a predefined reference group. For model-establishment experiments, the untreated control was used as the reference. For protection experiments, the AAPH group was used as the reference. When all pairwise comparisons were required, Tukey’s multiple comparisons test was used for multiple-comparison adjustment. For non-parametric comparisons, Kruskal–Wallis tests followed by Dunn’s multiple comparisons tests with adjustment for multiple comparisons were performed. Adjusted *p*-values were reported for multiple comparisons when applicable. Figures were generated using GraphPad Prism 8.0 (GraphPad Software, San Diego, CA, USA). Data are presented as mean ± standard deviation (SD) unless otherwise indicated. For box-and-whisker plots, the center line represents the median, the box represents the interquartile range (IQR), the whiskers indicate the range within 1.5 × IQR, and individual points beyond the whiskers were considered outliers. A value of *p* < 0.05 was considered statistically significant.

## 3. Results

### 3.1. Establishment of the AAPH-Induced Erythrocyte Oxidative Damage Model and Selection of Curcumin Concentration

**Figure 1:**
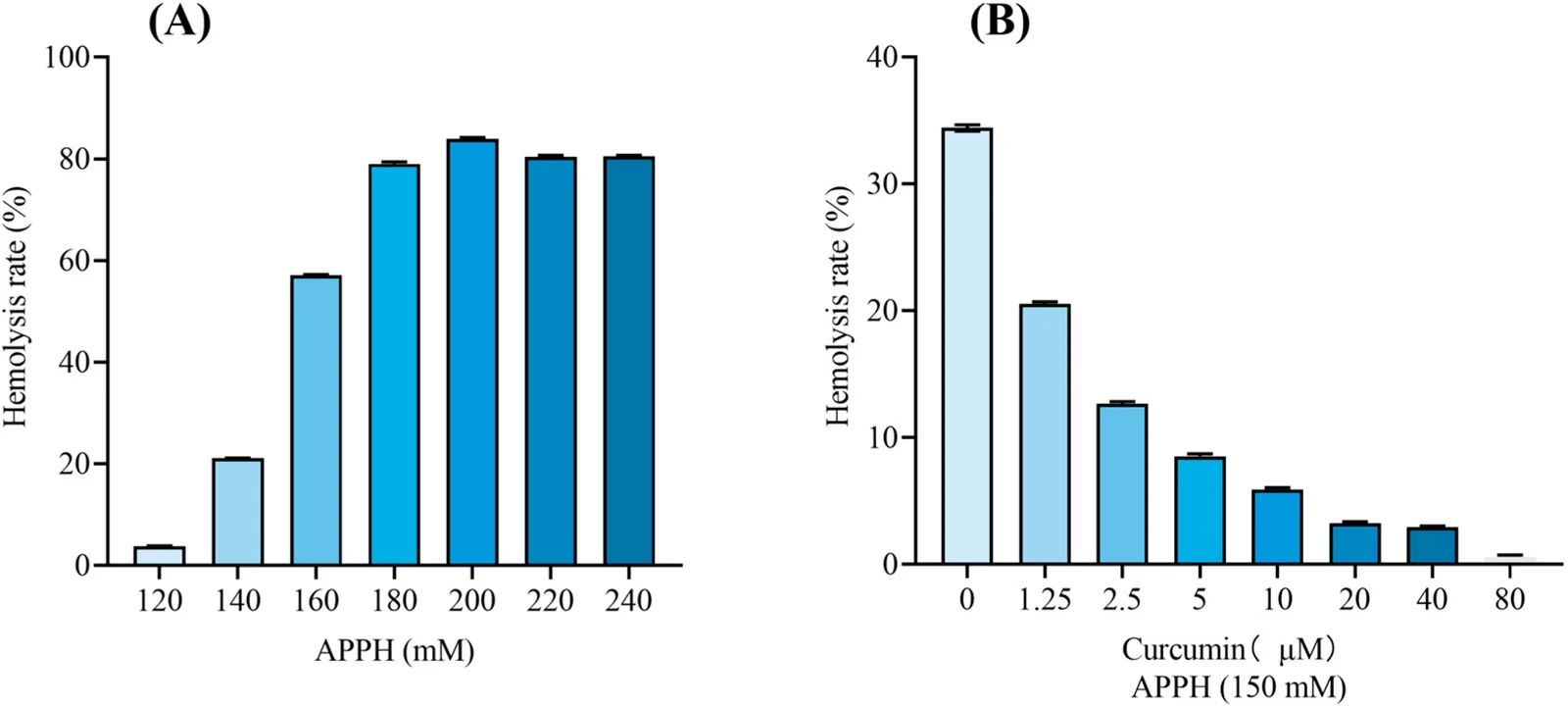
Effects of AAPH and curcumin pretreatment on erythrocyte hemolysis. (**A**) Hemolysis rates induced by different concentrations of AAPH. (**B**) Hemolysis rates of erythrocytes pretreated with curcumin at 1.25, 2.5, 5, 10, 20, 40, and 80 μM before exposure to 150 mM AAPH.

Hemolysis was first evaluated over a broad range of AAPH concentrations (120–240 mM) to identify an appropriate oxidative injury level for subsequent AFM analysis. A target hemolysis rate of 30–40% was predefined to provide sufficient oxidative damage while preserving an adequate number of intact erythrocytes for nanoscale characterization. As shown in Figure 1A, AAPH induced erythrocyte hemolysis in a concentration-dependent manner. At 140 mM, the hemolysis rate was 21.08%, indicating that the oxidative injury was relatively limited, which would reduce the dynamic range for evaluating the effects of curcumin pretreatment on AAPH-induced hemolysis. Increasing the AAPH concentration to 160 mM resulted in 57.09% hemolysis and generated a large amount of erythrocyte debris, which could interfere with the AFM measurements. At the concentrations from 180 to 240 mM, hemolysis further increased to approximately 80%, indicating extensive erythrocyte destruction. Therefore, 150 mM AAPH was selected as an intermediate concentration and further validated under the final experimental conditions. The validation experiment confirmed that 150 mM AAPH induced 34.42% hemolysis, which was within the predefined target range. Therefore, 150 mM AAPH was used to establish the oxidative injury model in subsequent experiments.

Curcumin pretreatment reduced AAPH-induced hemolysis in a concentration-dependent manner (Figure 1B). In the AAPH-only group, corresponding to 0 μM curcumin, the hemolysis rate reached 34.42%. In contrast, curcumin pretreatment markedly inhibited hemolysis across the concentration range, with hemolysis rates decreasing to 20.54%, 12.66%, 8.53%, 5.89%, 3.23%, 2.94%, and 0.61% at curcumin concentrations of 1.25, 2.5, 5, 10, 20, 40, and 80 μM, respectively. The curcumin-only group showed a hemolysis level comparable to that of the DMSO vehicle control, indicating that curcumin did not induce detectable hemolysis under the present conditions. Since curcumin was dissolved in DMSO, the concentration selection considered both protective efficacy and erythrocyte compatibility. The 80 μM treatment reduced AAPH-induced hemolysis from 34.42% to 0.61%, indicating sufficient protective efficacy. Therefore, 80 μM curcumin was selected for subsequent confocal microscopy and AFM experiments. These results are consistent with the AAPH oxidative damage model and indicate that 80 μM curcumin was within the concentration range without detectable hemolytic effects under the present experimental conditions.

### 3.2. Curcumin Preserves Cytoskeletal Organization and Nuclear Morphology

Because membrane skeleton integrity is closely related to erythrocyte mechanical function, confocal microscopy was used to examine F-actin and nuclear morphology after the AAPH injury and curcumin pretreatment.

**Figure 2:**
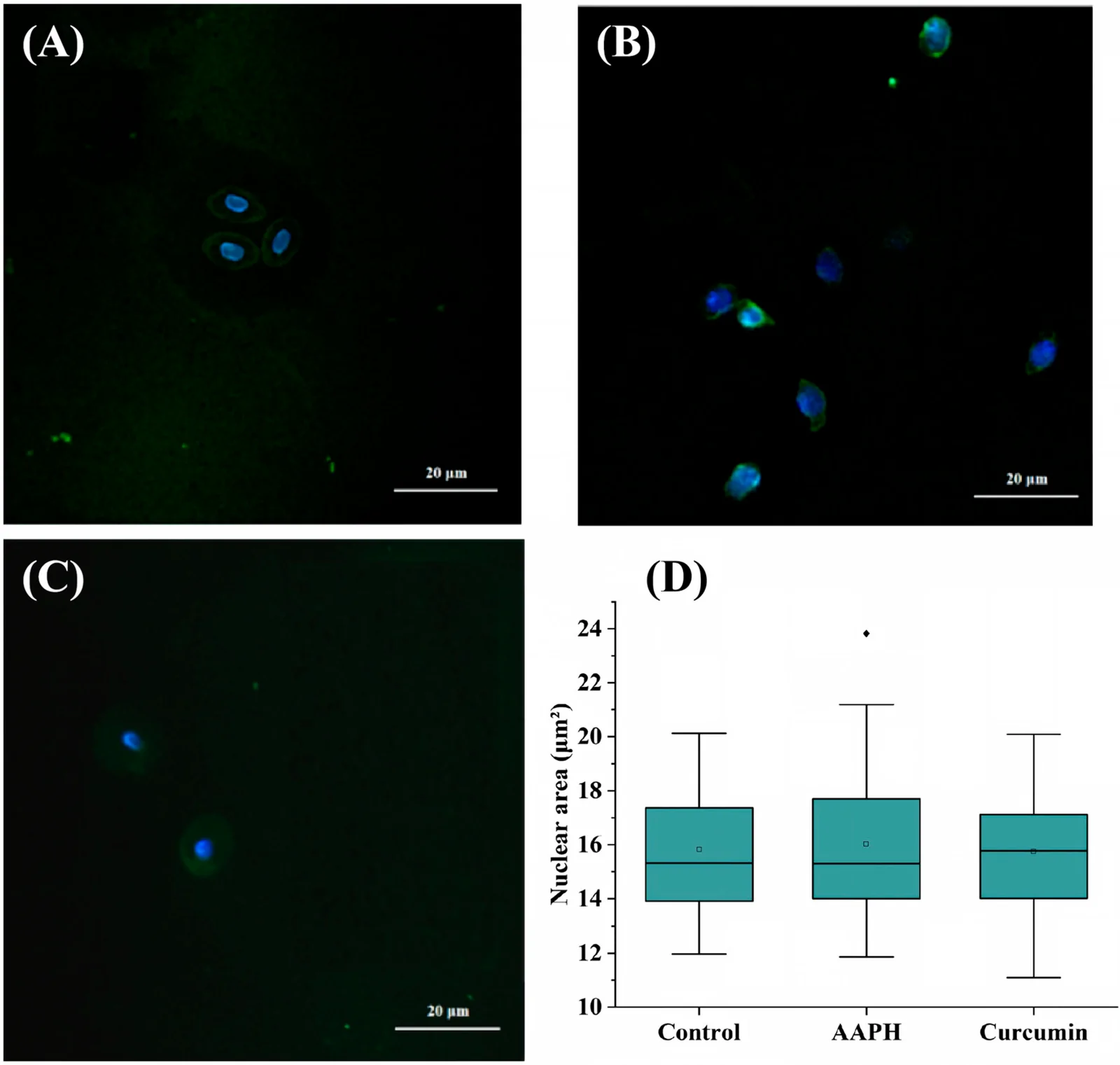
Confocal fluorescence images and quantitative analysis of chicken erythrocytes after AAPH injury and curcumin pretreatment. (**A**–**C**) Merged fluorescence images of the Control, AAPH-injured, and Curcumin-treated groups. (**D**) Box plots showing the nuclear area distribution among the three groups. Statistical analysis was performed using one-way ANOVA followed by Tukey’s multiple comparisons test, and adjusted *p*-values are shown. DAPI staining was used to visualize nuclei (blue fluorescence), and FITC-phalloidin staining was used to label F-actin cytoskeleton (green fluorescence). Scale bar = 20 μm.

As shown in Figure 2, confocal fluorescence imaging was used to evaluate the effects of AAPH-induced oxidative injury and curcumin pretreatment on cellular morphology and cytoskeletal organization. DAPI staining was used to visualize nuclei, while FITC-labelled phalloidin was used to label F-actin. As shown in Figure 2, the merged fluorescence images are presented in panels A–C, while panel D shows the box plots of cell number and cell area for the Control group, AAPH-injured group, and Curcumin-treated group.

In the control group (Figure 2A), F-actin exhibited a continuous and relatively uniform distribution, and cells maintained an intact oval-like morphology with nuclei showing regular shapes and clear boundaries. In contrast, after the AAPH exposure (Figure 2B), cells showed obvious shrinkage and deformation, accompanied by discontinuous and aggregated F-actin fluorescence. The nuclei also became shrunken and distorted, with irregular margins, indicating the pronounced oxidative damage to cellular structure. Notably, in the curcumin-treated group (Figure 2C), the F-actin network was better preserved compared with the AAPH group, and the extents of cellular shrinkage and nuclear deformation were partially alleviated. Although the cells tended to display a more rounded morphology rather than fully recovering the oval-like shape observed in the control group, curcumin pretreatment still provided protection against AAPH-induced structural damage. These observations indicate that AAPH exposure was associated with altered erythrocyte membrane-skeleton organization and nuclear morphology, whereas curcumin pretreatment partially preserved these structural features. This observation is consistent with previous studies reporting that curcumin possesses antioxidant activities [36,37,38]. Our confocal results provide subcellular evidence supporting the subsequent AFM-based mechanical characterization.

Quantitative analysis of Figure 2D further revealed the distribution of nuclear areas among the three groups. The nuclear areas were 15.83 ± 2.42 μm^2^ in the Control group, 16.02 ± 2.81 μm^2^ in the AAPH-injured group, and 15.74 ± 2.48 μm^2^ in the Curcumin-treated group. The AAPH-injured group showed a slight increase in the mean nuclear area compared with the Control group, whereas the Curcumin-treated group exhibited a value comparable to the Control group. The box plots demonstrated overlapping distributions among the three groups, suggesting that AAPH treatment caused only a minor alteration in nuclear area under the present experimental conditions. One-way ANOVA followed by Tukey’s multiple comparisons test showed no significant differences between the Control and AAPH groups (adjusted *p* = 0.9605), AAPH and Curcumin-treated groups (adjusted *p* = 0.9290), or Control and Curcumin-treated groups (adjusted *p* = 0.9924). These results indicate that, although AAPH induced obvious cytoskeletal disruption and cellular morphological changes, the nuclear area was relatively maintained, while curcumin pretreatment contributed to preserving cellular structural integrity.

### 3.3. Optimization of Short-Term Storage Conditions Before AFM Measurements

Before AFM characterization, a short-term storage condition was required to maintain erythrocyte morphology during the interval between oxidative treatment and AFM measurements. Since the oxidative injury experiment was completed after a 3 h reaction period, AFM measurements were performed within 8 h after treatment. Therefore, we first evaluated the morphological stability of untreated control erythrocytes under different storage conditions to identify an appropriate preservation environment that minimized storage-induced morphological changes.

**Figure 3:**
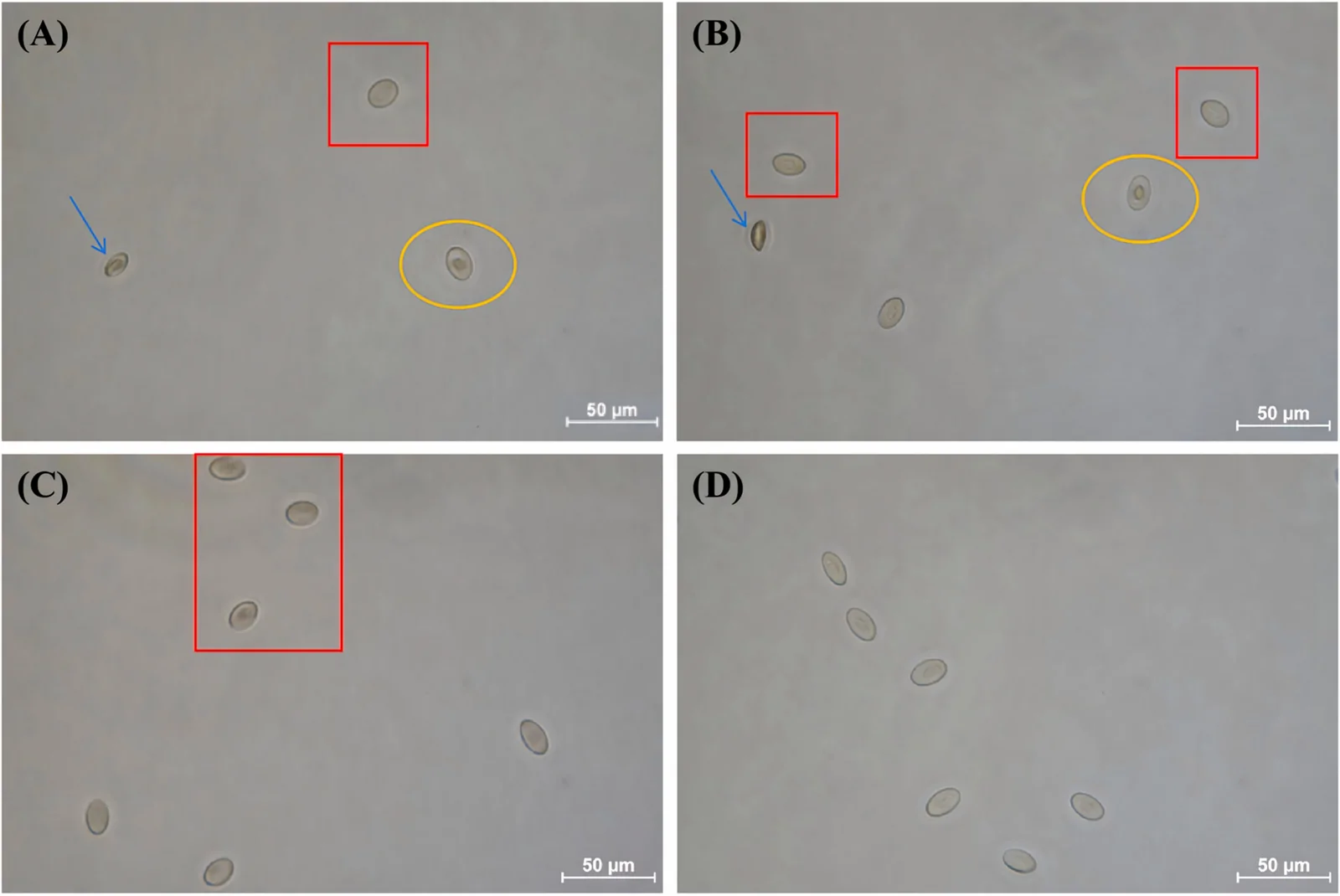
Morphological evaluation of chicken erythrocytes under different short-term storage conditions before AFM measurements. (**A**) PBS at 23 °C for 8 h; (**B**) PBS at 4 °C for 8 h; (**C**) Alsever’s solution at 23 °C for 8 h; (**D**) Alsever’s solution at 4 °C for 8 h. Red boxes indicate cell rounding, yellow circles indicate nuclear region protrusion, and blue arrows indicate severe cell deformation. Scale bar: 50 μm.

Control erythrocytes were stored in PBS or Alsever’s solution at 4 °C or 23 °C for 8 h, and their morphology was examined by inverted optical microscopy (Figure 3). In PBS at 23 °C (Figure 3A), erythrocytes showed obvious morphological instability, including severe deformation (blue arrow), cell rounding (red box), and protrusion of the nuclear region (yellow circle). Storage in PBS at 4 °C (Figure 3B) reduced the occurrence of severe deformation; however, some cells still exhibited rounded morphology and nuclear region protrusion, indicating that low temperature alone was insufficient to fully maintain the original cellular structure.

Compared with PBS, Alsever’s solution provided better morphological preservation. After storage in Alsever’s solution at 23 °C for 8 h (Figure 3C), most erythrocytes maintained relatively intact morphology, although several cells showed slight rounding (red box). When stored in Alsever’s solution at 4 °C (Figure 3D), erythrocytes exhibited clear contours and maintained a morphology close to that before storage, with no obvious cell rounding, nuclear region protrusion, or severe deformation.

These results demonstrate that Alsever’s solution at 4 °C provides the most suitable short-term storage condition for preserving erythrocyte morphology before AFM analysis. Because this condition maintained the structural stability of untreated erythrocytes within the 8 h measurement window, it was selected for subsequent AFM characterization of control, AAPH-treated, and curcumin-treated erythrocytes. Under this condition, the observed AFM differences are more likely to reflect treatment-induced structural alterations rather than storage-related artifacts. In this study, the preservation capability of Alsever’s solution at 4 °C was demonstrated within the 8 h experimental window. Therefore, we defined 8 h as the validated storage duration for maintaining erythrocytes suitable for subsequent morphology and nanomechanical analyses, rather than as a longer-term storage limit.

### 3.4. AFM Topography Characterization

**Figure 4:**
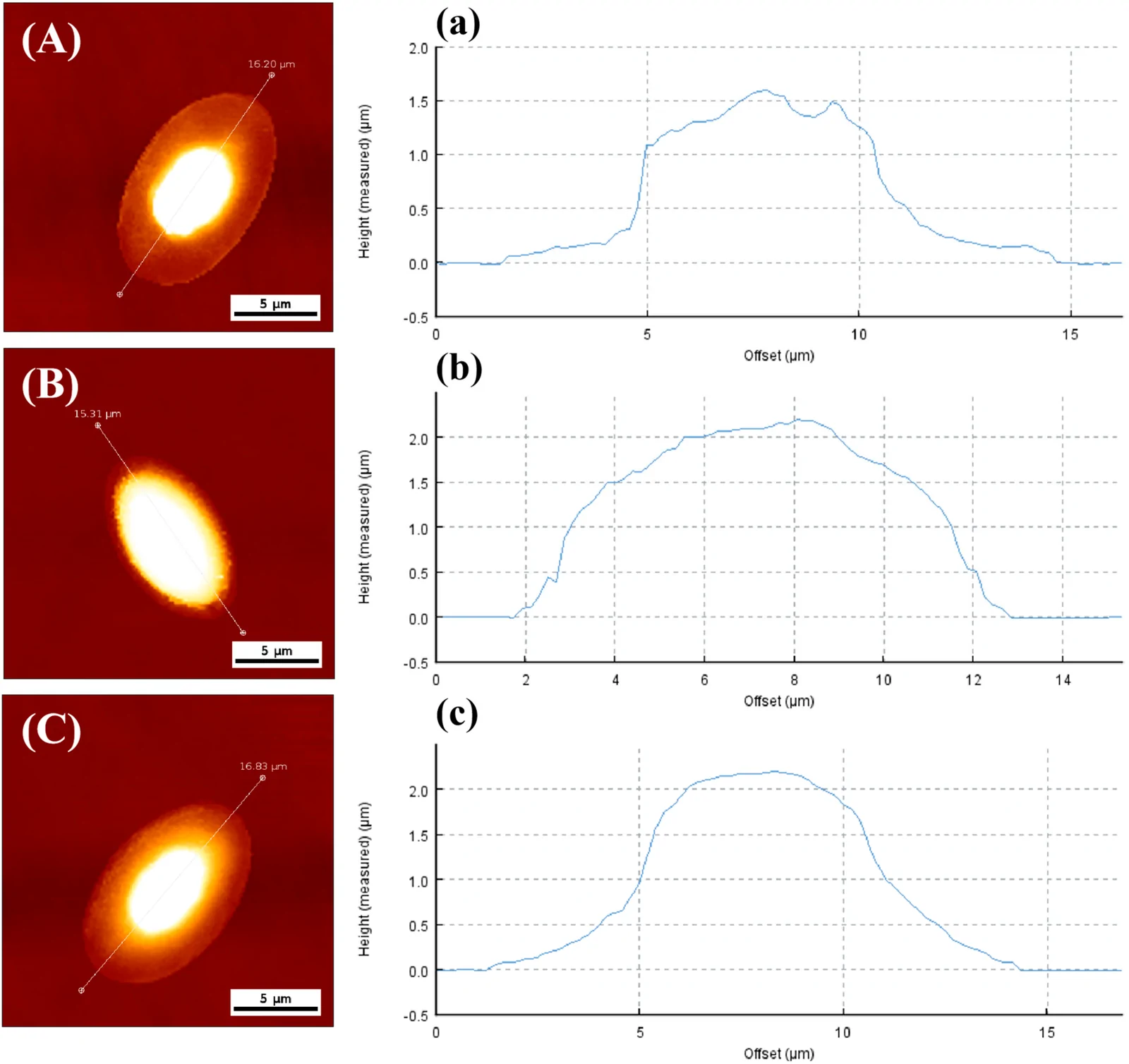
Representative AFM topography images and cross-sectional height profiles of chicken erythrocytes in different treatment groups. (**A**–**C**) AFM topography images of erythrocytes in the control, AAPH-treated, and curcumin-pretreated groups, respectively; (**a**–**c**) Cross-sectional height profiles corresponding to the line sections indicated in Panels (**A**–**C**), respectively. Scale bar: 5 μm.

Representative AFM topography images and height profiles were obtained to evaluate erythrocyte morphological changes in the control, AAPH and curcumin-treated groups (Figure 4).

Panels A–C show the representative AFM topography images from the control, AAPH-treated and curcumin-pretreated groups, respectively, and Panels a–c show the corresponding height profiles along the indicated line sections.

In the control group, chicken erythrocytes exhibited a typical elliptical disc-like morphology with a smooth surface, regular cell margin and centrally elevated nucleus. A characteristic annular depression was observed between the raised nuclear region and the elevated peripheral cytoplasmic rim (Figure 4A). The corresponding cross-sectional height profile showed that the height of the nuclear region was approximately 1.6 μm (Figure 4A). In contrast, the AAPH-treated erythrocytes retained an overall elliptical outline but displayed pronounced morphological alterations, including the disappearance of the annular depression and an overall increase in cell height. The nuclear region height increased to approximately 2.2 μm after AAPH treatment (Figure 4B,b). Compared with the AAPH-treated group, the erythrocytes in the curcumin-pretreated group showed a partial morphological improvement, with the partial restoration of the annular depression structure and the nuclear region height of approximately 2.1 μm (Figure 4C,c). These results indicate that the curcumin pretreatment partially alleviated the AAPH-induced morphological alterations but did not completely reverse the oxidative damage.

**Figure 5:**
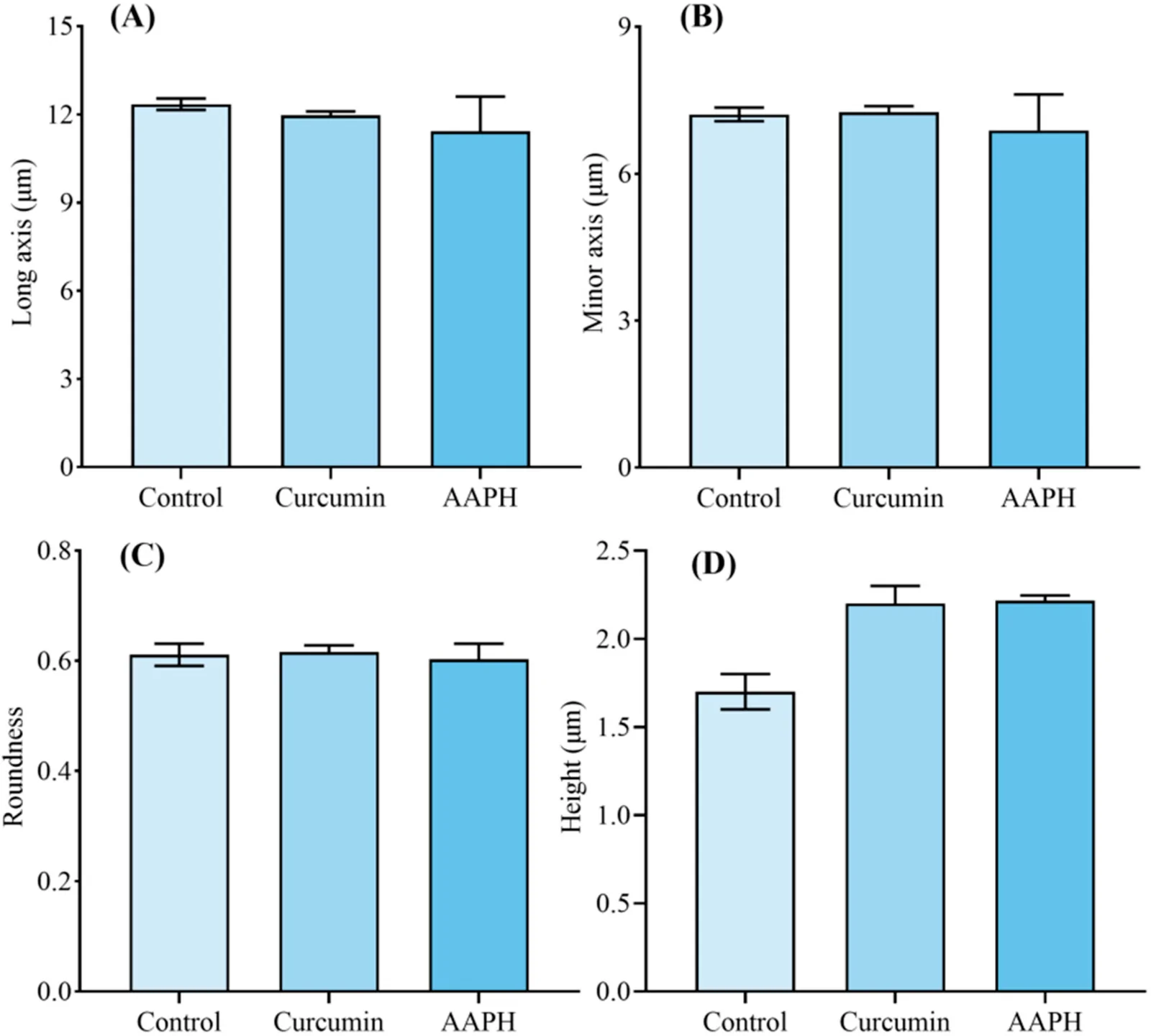
Statistical analysis of morphological parameters of chicken erythrocytes in different groups. (**A**) Long axis; (**B**) minor axis; (**C**) roundness; (**D**) height. Data are presented as mean ± SD. Statistical analysis was performed using one-way ANOVA followed by Tukey’s multiple comparisons test, and adjusted *p*-values are shown.

To further quantify the morphological changes of chicken erythrocytes in different treatment groups, four shape-related parameters, including long-axis length, minor-axis length, roundness, and maximum cell height, were statistically analyzed (Figure 5). As shown in Figure 5A,B, AAPH treatment reduced both the long- and minor-axis lengths of erythrocytes, from 12.06 μm and 7.36 μm in the control group to 11.43 μm and 6.88 μm, respectively, indicating AAPH-associated cell shrinkage. In contrast, the long- and minor-axis lengths in the curcumin-pretreated group were restored to 12.17 μm and 7.44 μm, respectively, showing no significant difference from the control group.

Roundness, calculated as the ratio of minor axis length to long axis length, was further analyzed to evaluate whether the elliptical morphology of erythrocytes was maintained (Figure 5C). The roundness values were comparable among the control, AAPH-treated and curcumin-pretreated groups with values of 0.611, 0.611 and 0.602, respectively. These results suggest that although the AAPH treatment altered the overall cell dimensions, it did not markedly disrupt the characteristic elliptical outline of chicken erythrocytes. Therefore, roundness alone is insufficient to fully assess the oxidative damage-induced morphological alterations and should be interpreted together with other dimensional and height-related parameters.

As shown in Figure 5D, the maximum height of the nuclear region increased markedly after AAPH treatment, from 1.78 μm in the control group to 2.22 μm, indicating the vertical deformation of erythrocytes under oxidative stress. In the curcumin-pretreated group, the maximum nuclear region height decreased to 2.10 μm, which was lower than that in the AAPH-treated group but remained higher than that in the control group.

Taken together, these results demonstrate that the AAPH-induced oxidative damage caused erythrocyte shrinkage along both the long and minor axes and increased the nuclear region height, while the overall elliptical shape was largely preserved. Curcumin pretreatment effectively restored the lateral dimensions of erythrocytes and partially attenuated the increase in the nuclear region height, suggesting that the curcumin pretreatment was associated with partial recovery of AAPH-induced morphological alterations. Statistical analysis of morphological parameters was performed using one-way ANOVA followed by Tukey’s multiple comparisons test. Among the four parameters, maximum cell height showed a significant difference among groups (one-way ANOVA, *p* = 0.0206), with the AAPH-treated group exhibiting a higher height compared with the Control group (adjusted *p* = 0.0276). However, no significant differences were observed between AAPH and Curcumin-treated groups (adjusted *p* = 0.7269) or between Control and Curcumin-treated groups (adjusted *p* = 0.0760). No significant differences were detected among groups for long-axis length (adjusted *p* = 0.3781–0.9598), minor-axis length (adjusted *p* = 0.3219–0.9682), or roundness (adjusted *p* = 0.9078–1.0000).

To further exploit the advantages of AFM for nanoscale surface characterization, we performed a quantitative analysis of surface roughness in the nuclear regions of cells from different treatment groups, in addition to the analysis of overall cell morphology and height. Unlike conventional optical microscopy, which mainly relies on two-dimensional optical contrast to visualize cell contours, AFM directly provides three-dimensional surface topography information and reveals local surface fluctuations at the nanoscale level. Therefore, the root mean square roughness (Rq) was used as a sensitive parameter to evaluate surface microstructural heterogeneity and local structural integrity of the nuclear region.

**Figure 6:**
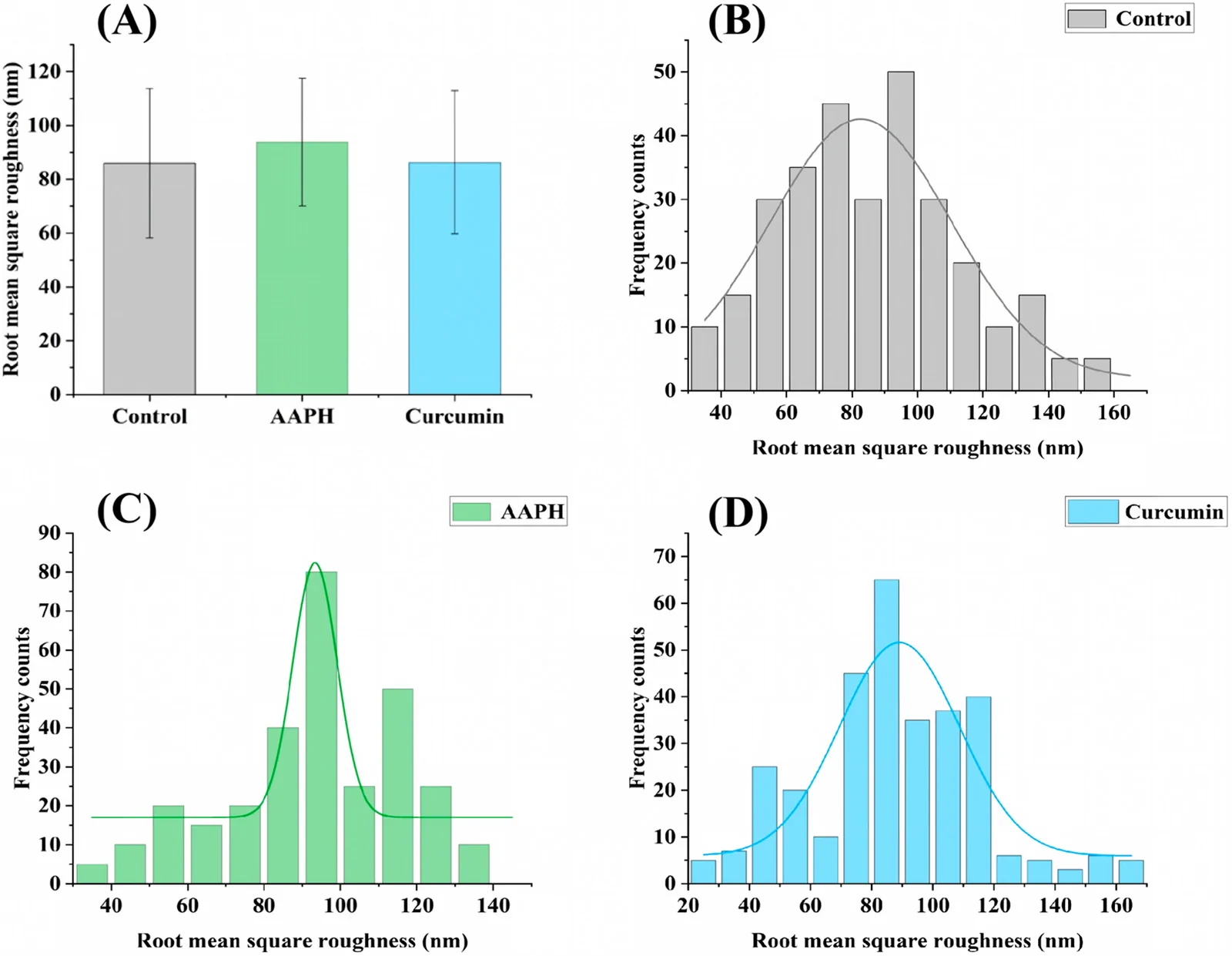
AFM-based quantitative analysis of nuclear surface roughness in chicken erythrocytes. (**A**) Representative histogram of root mean square roughness (Rq) values obtained from the nuclear regions of erythrocytes in the Control, AAPH-treated, and curcumin-treated groups. (**B**–**D**) Frequency distribution histograms of Rq values for the Control, AAPH-treated, and curcumin-treated groups, respectively. Data are presented as mean ± SD. Statistical analysis was performed using one-way ANOVA followed by Tukey’s multiple comparisons test, and adjusted *p*-values are shown.

The Rq values of 600 randomly selected nuclear regions from each group were statistically analyzed. The Rq histogram (Figure 6A) and Rq distribution frequency histograms (Figure 6B–D) were generated. The results showed that the Rq values were 85.93 ± 27.76 nm, 93.79 ± 23.77 nm, and 86.25 ± 26.62 nm in the Control, AAPH-treated, and curcumin-treated groups, respectively. Compared with the Control group, AAPH treatment increased the Rq value of the nuclear region, indicating enhanced nanoscale surface irregularity caused by oxidative stress. This change may reflect alterations in membrane-associated structural organization and local nanoscale heterogeneity after AAPH exposure.

Notably, the Rq distribution in the AAPH group shifted toward higher roughness values. After curcumin pretreatment, the Rq value was restored to a level close to that of the Control group. This result suggests that curcumin alleviated the surface structural abnormalities induced by oxidative damage and maintained nanoscale surface uniformity. Statistical analysis of nuclear surface roughness was performed using one-way ANOVA followed by Tukey’s multiple comparisons test. No significant differences were observed among the Control, AAPH-treated, and Curcumin-treated groups. Specifically, the AAPH group showed no significant difference compared with the Control group (adjusted *p* = 0.2291) or the Curcumin-treated group (adjusted *p* = 0.2571), while no significant difference was observed between the Control and Curcumin-treated groups (adjusted *p* = 0.9976).

These findings further demonstrate that AFM provides information beyond the cell size and morphology observable by optical microscopy. It can also reveal nanoscale surface structural changes that are difficult to detect using conventional optical methods. By combining morphological parameters, height measurements, and roughness analysis, AFM provides more comprehensive single-cell evidence for evaluating AAPH-induced cellular damage and curcumin-associated preservation.

### 3.5. AFM Nanomechanical Characterization

**Figure 7:**
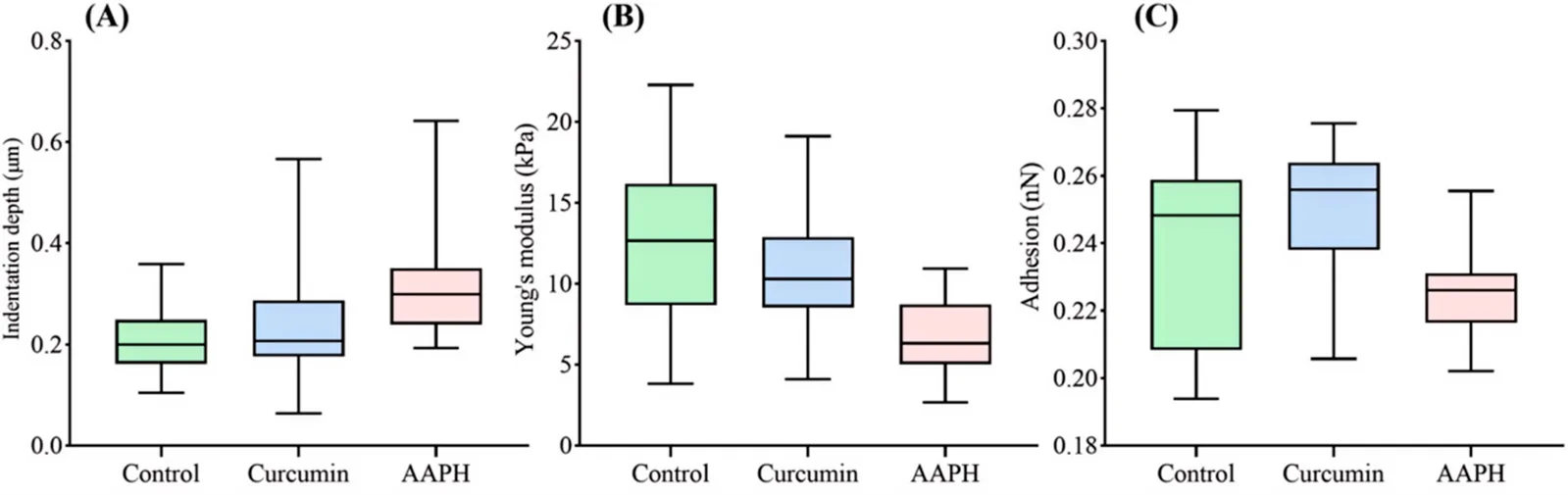
Box-and-whisker plots of the nanomechanical properties of chicken erythrocytes measured by AFM. (**A**) Indentation depth; (**B**) Young’s modulus; (**C**) adhesion force. The center line represents the median, the box indicates the interquartile range (IQR), the whiskers represent values within 1.5 × IQR, and points beyond the whiskers are shown as outliers.

Erythrocyte mechanical properties are closely linked to membrane integrity, deformability and the oxygen transport function. Therefore, the indentation depth, apparent Young’s modulus, and adhesion force of the nuclear region were analyzed to evaluate the mechanical consequences of AAPH-induced injury and curcumin-associated preservation (Figure 7).

As shown in Figure 7A, the indentation depth of the nuclear region differed significantly among the three groups. Compared with the control group (0.21 ± 0.06 μm), erythrocytes exposed to AAPH exhibited a greater indentation depth (0.32 ± 0.11 μm), indicating reduced resistance to external deformation under the present AFM loading conditions. Curcumin pretreatment partially attenuated this increase, resulting in a lower indentation depth (0.24 ± 0.10 μm) that approached the control level. Dunn’s multiple comparisons test showed that the AAPH group exhibited significantly higher indentation depth than both the control group and curcumin-pretreated group (adjusted *p* < 0.001), whereas no significant difference was observed between the control and curcumin-pretreated groups (adjusted *p* = 0.174).

Consistent with the indentation results, the apparent Young’s modulus of the nuclear region (Figure 7B) was significantly reduced following AAPH treatment, decreasing from 12.34 ± 4.51 kPa in the control group to 6.84 ± 2.35 kPa. This decrease suggests the weakened resistance of the measured nuclear region to indentation after oxidative injury. Curcumin pretreatment partially restored the mechanical response, yielding a Young’s modulus of 10.97 ± 3.37 kPa. Although the control group exhibited a relatively broader distribution of Young’s modulus values, reflecting the intrinsic heterogeneity in erythrocyte mechanical properties, the overall trend indicated that the curcumin mitigated AAPH-induced softening of the measured region. Dunn’s multiple comparisons test indicated that the apparent Young’s modulus was significantly lower in the AAPH group than in both the control and curcumin-pretreated groups (adjusted *p* < 0.001), while no significant difference was detected between the control and curcumin-pretreated groups (adjusted *p* = 0.159).

The adhesion force at the nuclear region is presented in Figure 7C. The control group exhibited a relatively broad distribution of adhesion force values (0.239 ± 0.266 nN), reflecting the intrinsic nanoscale heterogeneity of erythrocyte surface properties. Compared with the control group, AAPH treatment significantly reduced the adhesion force to 0.225 ± 0.089 nN, indicating altered surface interaction characteristics after oxidative stress exposure. Since AFM-derived adhesion force represents the combined contribution of surface molecular composition, membrane organization, hydration effects, and probe–cell interactions, the observed reduction should be considered as an overall change in nanoscale interfacial properties rather than a direct indication of a specific molecular alteration. Together with the increased indentation depth, decreased apparent Young’s modulus, and disrupted F-actin organization observed after AAPH treatment, these results suggest that oxidative stress impaired the structural and mechanical characteristics of the erythrocyte membrane–cytoskeleton system.

Curcumin pretreatment increased the adhesion force to 0.248 ± 0.020 nN, which was higher than that of the AAPH-treated group and showed partial recovery toward the control level. Dunn’s multiple comparisons test showed that adhesion force differed significantly between the AAPH group and the control group (adjusted *p* = 0.0017), as well as between the AAPH group and the curcumin-pretreated group (adjusted *p* < 0.001). The curcumin-pretreated group also differed from the control group (adjusted *p* = 0.0183). This recovery, together with the reduced hemolysis, improved F-actin organization, and partially restored cellular morphology observed after curcumin treatment, indicates that curcumin treatment was associated with reduced oxidative stress-induced alterations in erythrocyte surface mechanical properties. Although the adhesion force value itself reflects a composite nanoscale interaction parameter rather than a single membrane component, its recovery provides additional nanomechanical evidence supporting the association between curcumin treatment and preservation of erythrocyte structural integrity.

## 4. Discussion

AAPH-induced oxidative stress produced a coordinated injury phenotype in chicken erythrocytes, including hemolysis, cytoskeletal disruption, nuclear shrinkage, cell shrinkage and altered nanomechanical properties. These changes are consistent with oxidative stress-induced disruption of membrane integrity and cytoskeletal organization, which are critical for maintaining erythrocyte morphology and mechanical stability. In the present AFM measurements, AAPH exposure increased the indentation depth of the nuclear region while decreasing its apparent Young’s modulus, suggesting that oxidative injury compromised the mechanical stiffness and deformation resistance of the nuclear region. Because AFM measurements were performed on intact erythrocytes, the apparent Young’s modulus obtained from nuclear-region force curves reflects the integrated mechanical contribution of the nucleus, surrounding cytoskeleton, membrane, and cellular geometry. Therefore, the observed decrease in apparent Young’s modulus should be interpreted as an alteration of the local mechanical response of the nuclear region within intact erythrocytes rather than definitive evidence of isolated intrinsic nuclear softening. Meanwhile, the decreased adhesion force may reflect altered nanoscale surface properties caused by changes in membrane organization, molecular composition and probe–cell interactions.

Importantly, AFM-derived morphological and nanomechanical changes were evaluated in the context of AAPH-induced oxidative injury, while potential influences from nonspecific chemical damage, mechanical stress, and storage-related alterations were minimized through standardized experimental conditions. The oxidative stress association was supported by the AAPH model, hemolysis analysis, cytoskeletal assessment, and curcumin-associated protection. All groups were processed under identical storage and AFM measurement conditions, and Alsever’s solution at 4 °C was used to preserve erythrocyte morphology during the AFM analysis period.

Curcumin pretreatment strongly reduced hemolysis and partially preserved F-actin and nuclear morphology. This structural preservation is consistent with previous reports describing the biological activities of curcumin [36,37,38]. Because curcumin possesses limited aqueous solubility and intrinsic fluorescence, potential effects related to aggregation and optical interference were carefully considered. In this study, curcumin fluorescence was not used as an imaging signal; instead, cellular morphology was assessed using FITC-phalloidin and DAPI staining, together with AFM-based structural and nanomechanical measurements. Importantly, AFM showed that these biochemical and structural improvements were accompanied by the recovery of nanoscale morphology and mechanical parameters, indicating that curcumin helps maintain membrane mechanical homeostasis under AAPH-induced oxidative stress.

The optimization of storage conditions is a methodological contribution of this study. The oxidatively damaged erythrocytes were unsuitable for AFM analysis after the storage in PBS at room temperature because of severe rupture and debris formation. Alsever’s solution at 4 °C preserved cell morphology most effectively among the tested conditions. The reason for selecting Alsever’s solution at 4 °C was that the combination of an isotonic preservation environment and low temperature minimized storage-associated deformation and maintained cells in a condition suitable for AFM measurement. However, because the present study focused on the short interval required for AFM preparation, longer storage periods were not evaluated.

In addition, systematic AFM-based investigation of AAPH-treated chicken erythrocytes remains limited, particularly for integrating oxidative injury, single-cell morphology and nanomechanical characterization. Therefore, the value of this study is not only the application of established AFM approaches, but also the development of an optimized workflow for measuring mechanically fragile, non-adherent blood cells under oxidative stress conditions.

The analytical strength of this study lies in the integration of hemolysis assays, confocal imaging and AFM. The hemolysis assays provided evidence of global membrane damage, while confocal microscopy revealed alterations in nuclear and cytoskeletal structures. Using liquid-phase QI mode, AFM further enabled quantitative assessment of erythrocyte morphology and nuclear nanomechanical properties at the single-cell level. Together, these methods provide complementary evidence for curcumin-associated structural and mechanical preservation and establish a practical workflow for evaluating oxidative damage-associated alterations in fragile nucleated erythrocytes.

## 5. Conclusions

This study combined hemolysis assays, confocal microscopy, and AFM to characterize AAPH-associated structural alterations and curcumin-associated protection in chicken erythrocytes. AAPH induced moderate hemolysis, cytoskeletal disruption, nuclear shrinkage, and erythrocyte morphological alterations. These structural changes were accompanied by altered nuclear nanomechanical properties, including increased indentation depth and reduced apparent Young’s modulus and adhesion force at the nuclear region, demonstrating compromised nuclear mechanical stability under oxidative stress. Curcumin pretreatment attenuated hemolysis, partially preserved the cytoskeletal and nuclear architecture, and partially restored the AFM-derived topographical and nanomechanical properties. In addition, storage in Alsever’s solution at 4 °C was identified as the best preservation condition among the tested conditions for the AFM analysis of oxidatively damaged erythrocytes. Collectively, these findings demonstrate that AFM, in combination with conventional biological assays, provides a quantitative single-cell analytical workflow for assessing oxidative stress-associated structural remodeling and curcumin-associated changes in fragile blood cells.

## Data Availability

The raw data supporting the conclusions of this article will be made available by the authors on request.

## Abbreviations

The following abbreviations are used in this manuscript:

AFM	atomic force microscopy
AAPH	2,2′-azobis(2-methylpropionamidine) dihydrochloride
DMSO	dimethyl sulfoxide
QI	quantitative imaging
SD	standard deviation
